# Problematic Online Gaming and Gambling Among First-Year University Students: Identification of Profiles and Psychological Vulnerabilities

**DOI:** 10.1007/s10899-026-10476-5

**Published:** 2026-03-04

**Authors:** Roser Granero, Fernando Fernández-Aranda, Zsolt Demetrovics, Susana Jiménez-Murcia

**Affiliations:** 1 Department of Psychobiology and Methodology, Universitat Autònoma de Barcelona - UAB, Barcelona, Spain; 2 Ciber Fisiopatología Obesidad y Nutrición (CIBERobn), Instituto Salud Carlos III, Madrid, Spain; 3 Group of Psychoneurobiology of Eating and Addictive Behaviours, Neuroscience Program, Institut d’Investigació Biomèdica de Bellvitge - IDIBELL, L’Hospitalet de Llobregat, Spain; 4 Department of Clinical Psychology, Hospital Universitari de Bellvitge, L’Hospitalet de Llobregat, Spain; 5 Department of Clinical Sciences, School of Medicine and Health Sciences, Universitat de Barcelona – UB, L’Hospitalet de Llobregat, Spain; 6 College of Education, Psychology and Social Work, Flinders University Institute for Mental Health and Wellbeing, Flinders University, Bedford Park, SA Australia; 7 Institute of Psychology, ELTE Eötvös Loránd University, Budapest, Hungary; 8 Centre of Excellence in Responsible Gaming, University of Gibraltar, Gibraltar, Gibraltar

**Keywords:** Internet Gaming, Online Gambling, University Students, Online Social Networking, Path analysis

## Abstract

**Background and aims:**

Problematic engagement in online gaming and gambling has been linked to a range of cognitive, emotional, and behavioral risk factors, yet the mechanisms underlying the severity of these behaviors remain underexplored within high-risk populations such as first-year university students. This study aimed to identify variables associated with internet gaming disorder (IGD) and online gambling disorder (OGD), and to examine potential mediating mechanisms influencing the severity of these addictive behaviors through path analysis.

**Methods:**

A total of 273 first-year undergraduate students participated in the study. IGD and OGD measures, cognitive distortions, impulsivity, emotion regulation, risk of social media and internet use and psychological distress were analyzed.

**Results:**

32.6% (95% confidence interval [CI]: 27.0% to 38.2%) reported problematic gaming use and 1.8% (95%CI: 0.2% to 3.4%) met criteria for IGD. Problematic OGD was identified in 7.0% (95%CI: 3.9% to 10.0%) participants, and 2.2% (95%CI: 0.5% to 3.9%) met criteria for OGD. Path analysis showed that: (a) IGD severity was primarily associated with male gender, higher use of social media and the internet, cognitive biases, and difficulties in emotion regulation; (b) OGD severity was mainly associated with male gender and gambling-related cognitive biases; and (c) the relationships of the students’ gender and age with IGD and OGD severity were mediated by cognitive biases, impulsivity, emotion dysregulation, and use of social media and the internet.

**Conclusions:**

Cognitive processes, emotional mechanisms, male gender and frequent social media use constitute key targets for early detection and preventive interventions among first-year university students.

## Introduction

Over the past two decades, digital technologies have reshaped leisure, social interaction, and consumption among adolescents and young adults. Widespread Internet access and mobile device availability have fuelled the growth of engaging online activities, including (multiplayer) video games, online gambling, social media interaction, streaming services, virtual reality experiences, and online education. Although most individuals use online platforms for recreational and professional activities without related harms, evidence increasingly points to the risks of problematic or addictive use (King & Delfabbro, 2019; Király et al., 2023), especially among highly vulnerable groups such as late adolescents and young adults (Dienlin & Johannes, 2020; Haddock et al., 2022). Early university students are a population at heightened risk (Metin-Orta & Demirtepe-Saygılı, 2023; Sánchez-Fernández & Borda-Mas, 2023).

Within the broader framework of online engagement, Internet Gaming Disorder (IGD) and Online Gambling Disorder (OGD) have emerged as particularly salient behavioral addictions (Brand et al., 2025a, 2025b; Crane et al., 2025; Katz et al., 2024; Petry et al., 2018; Potenza, 2017) among young adults. IGD is characterized by persistent and recurrent engagement in online gaming, leading to significant impairment or distress, such as preoccupation with gaming, loss of control, and negative consequences in social, academic, or occupational functioning. Gaming disorder is recognized as an official diagnosis in the International Classification of Diseases (ICD-11) by the World Health Organization (World Health Organization, 2019) and is included in Section III of the Diagnostic and Statistical Manual of Mental Disorders, Fifth Edition (DSM-5) as a condition warranting further empirical investigation (American Psychiatric Association, 2013). OGD involves persistent and problematic engagement in online gambling activities, with symptoms including preoccupation with gambling, inability to limit time or money spent, and continuation of gambling despite adverse social, financial, or emotional consequences. OGD represents a subtype of gambling disorder that takes place through websites or mobile applications rather than in traditional in-person settings. Importantly, gambling disorder is classified in the DSM-5 under ‘Substance-Related and Addictive Disorders’ underscoring that addictive behaviors may arise not only from substances but also from certain rewarding activities (Moreira et al., 2023). OGD has gained growing visibility as a high-risk behavior among young adults owing to its ease of access, constant availability, anonymity, and the wide variety of games offered online (Barrera-Algarín & Vázquez-Fernández, 2021; Calado & Griffiths, 2016; Ghelfi et al., 2024).

Studies have reported significant functional impairment across multiple life domains for both IGD (Halley & Griffiths, 2015; Männikkö et al., 2015) and OGD (Ford & Håkansson, 2020; Mora-Salgueiro et al., 2021). Impairments include reduced academic and/or occupational performance, social withdrawal, conflicts with family members, increased risk of comorbid mental health problems and decreased psychological well-being. Research on vulnerability factors has identified a set of cognitive, emotional, and behavioral variables that contribute to the development and maintenance of these non-substance-related addictions. Among the most prominent are problematic-excessive use of social media and internet (Andreassen et al., 2017; Marino et al., 2018; Montag et al., 2024; Moretta et al., 2022), impulsivity (Ioannidis et al., 2019; King et al., 2019), emotion regulation deficits (Estupiñá et al., 2024; Rogier & Velotti, 2018); and cognitive distortions related with the gambling activity (Armstrong et al., 2020). Moreover, in recent years, the monetization of video games through mechanisms such as loot boxes has introduced additional risks. These randomized, paid rewards not only resemble gambling but have been shown to mediate the relationship between IGD and OGD, increasing the likelihood of migration from gaming to gambling behaviors and potentially leading to significant financial harm (McCaffrey, 2023; Palmer et al., 2025).

Sociodemographic variables, notably male gender and younger age, have also been consistently identified as predictors of higher risk of IGD and OGD, likely reflecting sociocultural influences, motivational factors, and patterns of digital socialization (Mihara & Higuchi, 2017; Stevens et al., 2021). Early adulthood (≈18–25 years) has received considerable attention in scientific research, as it represents a critical developmental period marked by heightened vulnerability to the onset and progression of a wide range of mental health disorders (Sheldon et al., 2021; Solmi et al., 2022), including Internet-related disorders (Wang et al., 2022). During this developmental stage, functional impairments and associated risks may be particularly severe and enduring, as young adults continue to develop prefrontal circuits and neurocognitive processes, strengthen self-regulation, refine decision-making and coping skills, and undergo social maturation (Wood et al., 2018).

Within the young adult population, first-year university students represent a key at-risk group for IGD and OGD due to the unique psychosocial challenges they face, including increased autonomy, separation from the family setting, exposure to new social environments, and heightened academic and social pressures (Bastien et al., 2025; Li et al., 2025). Studies have observed that these multiple challenges may heighten psychological vulnerability and promote reliance on online activities as a means of emotional regulation or escape (Kumar & Mondal, 2018; Lipson et al., 2019; Normala & Azlini, 2018). The situation is further complicated by the multifaceted ways in which university students engage with the Internet, spanning academic activities, social interaction, entertainment, and access to everyday services. While such pervasive use is common across populations, it places students in a context of near-constant online exposure, increasing the likelihood of engagement in potentially problematic online behaviors. These behaviors may interfere with academic and occupational functioning, hinder the development of healthy social relationships, and exacerbate emotional vulnerabilities (Rouvinen et al., 2021).

Current meta-analysis estimates the global prevalence of internet addiction among university students at around 42%, with considerable variation according to gender, income level, geographic region, pandemic period, measurement instruments, and type of addiction (Liu et al., 2025). Among the various forms of Internet-related problems, IGD and OGD have shown the most pronounced increases among young university students, with point estimates around 5% for IGD (Sánchez-Fernández & Borda-Mas, 2023) and 0.5% for OGD (Grant et al., 2019). Although both conditions have been associated with a range of functional impairments, the scientific literature still shows significant gaps, as much of the existing research has focused on the general population or clinical samples without sufficiently addressing this high-risk target group. Further scientific evidence is needed to characterize the profiles associated with IGD and OGD among first-year university students and to elucidate the complex interrelationships among the various psychosocial vulnerability factors.

In this context, the present study aims to: (a) estimate the presence and severity of IGD and OGD in a sample of first-year university students; (b) identify the cognitive, emotional, behavioral, and sociodemographic factors associated with these behaviors; and (c) analyze the mediating mechanisms linking these variables to the severity of online gaming and gambling, in order to provide evidence that refines risk profiles and informs early prevention and intervention strategies in university settings.

Based on the available evidence, the following empirical hypotheses are proposed: (a) the severity of IGD and OGD will be positively associated with impulsivity, cognitive distortions, difficulties in emotion regulation, and problematic use of social media and the internet; (b) male gender and younger age will be related to higher levels of severity in these behaviors; and (c) these associations will be partially mediated by psychological variables, such that the effects of gender and age on IGD and OGD will be partly explained by higher levels of cognitive biases, impulsivity, emotion regulation difficulties, and risk of addiction to social networks and the Internet.

## Methods

### Participants

The dataset analyzed in this study was drawn from a broader longitudinal project aimed at examining risk factors for problematic online gaming and gambling, as well as predictors of clinical severity and treatment outcomes. Recruitment took place at the Autonomous University of Barcelona (Spain). Eligible participants were first-year psychology undergraduates aged 18–25 years. To ensure sample homogeneity and reduce variability associated with academic trajectory, socio-demographic background, or prior university experience, data collection was restricted to a single program and cohort year.

Data were collected in classroom sessions between May 2024 and February 2025 from approximately 75% of first-year psychology students across the 2023–2024 and 2024–2025 cohorts. Assessments, lasting about 60 min in groups of around 20 students, were administered by senior researchers, with participation entirely voluntary and without incentives.

### Measures

Diagnostic Questionnaire for IGD. This nine-item self-report instrument operationalizes the DSM-5 criteria for Internet Gaming Disorder (IGD; American Psychiatric Association, 2013) as further specified by Petry et al. (2014). It assesses the nine IGD symptoms—preoccupation, withdrawal, tolerance, loss of control, neglect of other activities, continued use despite negative consequences, deception, gaming to escape negative moods, and jeopardizing relationships or responsibilities—using a dichotomous (yes/no) format, with five or more positive responses indicating a potential diagnosis. In the present sample, the questionnaire showed acceptable internal consistency (Cronbach’s α = 0.720).

Diagnostic Questionnaire for Gambling Disorder. This self-report instrument is a Spanish adaptation (Jiménez-Murcia et al., 2009) of the original Diagnostic Questionnaire for Gambling Disorder developed by Stinchfield (2003), designed to assess gambling pathology according to DSM-IV-TR (American Psychiatric Association, 2010) and DSM-5 (American Psychiatric Association, 2013) criteria. Nine dichotomous (yes/no) items capture behavioral and cognitive symptoms of disordered gambling, and in this study, were completed with reference to online gambling behaviors. Endorsement of four or more items indicates a potential gambling disorder, and the measure showed satisfactory internal consistency in the present sample (Cronbach’s α = 0.752).

Scale of Risk of Addiction to Social Networks and Internet (RASNI). The RASNI is a 29-item self-report instrument designed to assess the intensity and problematic patterns of social network and Internet use. Items are rated on a Likert-type scale and organized into four empirically and theoretically derived factors: addiction severity (compulsive and excessive use), social use (frequency and intensity of online interactions), atypical behaviors (e.g., participation in interest-based groups, virtual or role-playing games, online sexual activities), and nomophobia (anxiety or distress when a mobile device or Internet access is unavailable). In this study, the validated Spanish version (ERA-RSI; Peris et al., 2018) was used. The scale demonstrated satisfactory internal consistency in the present sample, with Cronbach’s α = 0.842.

Gambling Related Cognitions Scale (GRCS). The GRCS is a 23-item self-report instrument initially developed to assess cognitive biases and distorted beliefs related to gambling (Raylu & Oei, 2004). The GRCS items are rated on a Likert-type scale and organized into five theoretically derived dimensions (interpretative bias, illusion of control, predictive control, gambling-related expectancies and perceived inability to stop gambling). For this study, we used the validated Spanish version (Del Prete et al., 2017), which demonstrates psychometric properties comparable to the original scale and other cross-cultural adaptations (Tang & Oei, 2011). GRCS scores in relation to IGD should not be interpreted as a direct measure of gaming-specific cognitions, but rather as an indicator of gambling-related cognitive distortions that may partially overlap with maladaptive cognitive processes associated with problematic gaming, particularly among young populations exposed to increasingly gamblified gaming environments. Although the GRCS items explicitly refer to gambling activities, several studies have examined the convergence of shared cognitive biases between problematic gambling and gaming, drawing on video game contexts that frequently involve elements of skill acquisition and learning from losses. For instance, Sanmartín and colleagues compared problematic gamblers with video gamers who accessed loot boxes (both purchasers and openers) and identified shared cognitive biases—specifically illusion of control and predictive control—as measured by the GRCS, even after controlling for overlap between groups (Sanmartín et al., 2024). Furthermore, the study found that problematic gamblers, compared with loot box purchasers, did not report statistically significant differences on most GRCS dimensions, including illusion of control, predictive control, interpretative biases, and gambling-related expectancies. In the current sample, the GRCS showed excellent internal consistency, with Cronbach’s α ranging from 0.779 (illusion of control) to 0.886 (interpretative bias) and α = 0.946 for the total scale.

Impulsive Behavior Scale (UPPS-P). This self-report includes 59 items assessing five facets of impulsivity (lack of premeditation, lack of perseverance, sensation seeking, negative urgency, and positive urgency), each linked to addictive and externalizing behaviors (Whiteside & Lynam, 2001). A global impulsivity score can also be computed by summing all items. In this study, the validated Spanish version was used (Verdejo-García et al., 2010), which showed excellent internal consistency in the current sample (α = 0.946).

Difficulties in Emotion Regulation Scale (DERS). The DERS is a 36-item self-report measure assessing individual differences in emotion regulation across six dimensions: lack of emotional awareness, lack of clarity, non-acceptance of emotions, difficulties engaging in goal-directed behavior, limited access to regulation strategies, and impulse control difficulties (Gratz & Roemer, 2004). A total score provides a comprehensive index of overall emotion regulation difficulties. In this study, the validated Spanish version was used (Hervás & Jódar, 2008; Wolz et al., 2015), showing excellent internal consistency in the current sample (α = 0.938).

Symptom Checklist-Revised (SCL-90-R). The 90-item SCL-90-R (Derogatis, 1999, 2002) assesses psychological symptoms across nine dimensions and provides three global distress indices. In this study, the validated Spanish version was used, focusing on the Global Severity Index (GSI) and Positive Symptom Total (PST) as overall indicators of symptom severity and breadth. Both indices demonstrated excellent internal consistency in the current sample (α = 0.975).

Other sociodemographic information. In addition to the primary measures, data were collected on participants’ gender, age, country of origin (born in Spain vs. other), and socioeconomic status (SES). SES was assessed using the Hollingshead index, which combines educational attainment and occupational status of household members into a composite social position score (Hollingshead, 2011). This standardized index provides a consistent measure of family socioeconomic background, enabling comparisons across studies.

### Ethical Considerations

The study adhered to the Declaration of Helsinki and was approved by the Research Ethics Committee of the Autonomous University of Barcelona (CEEAH 6564; January 19, 2024). All participants gave written informed consent, confirming voluntary participation and the right to withdraw at any time, with no financial or material compensation provided.

### Statistical Analyses

Statistical analysis was conducted with Stata19 for Windows (StataCorp, 2025). First, comparisons were conducted between participants with problematic behavior related to IGD or OGD—defined as meeting at least one DSM-5 criterion for the respective disorder—and those without such problems. Chi-square tests were used for categorical variables, and independent-samples t-tests were applied for continuous variables. Effect sizes were calculated using Cramer’s-V for categorical associations and Cohen’s-d for mean differences. Given the relatively small size of the group exhibiting problematic behavior, both statistical significance and a minimum of moderate effect size were considered relevant for interpreting the results (C-V > 0.20 or |d|> 0.50; Cohen, 1998). In addition, to control for Type I error inflation due to multiple statistical testing, the Finner stepwise adjustment procedure (Finner & Roters, 2002) was applied, providing more powerful family-wise error rate control than traditional corrections such as Bonferroni.

Path analyses were performed using structural equation modeling (SEM), which allows the simultaneous estimation of multiple relationships and the assessment of direct and indirect effects. SEM was used to examine how sociodemographic variables (gender and age), cognitive biases related to the gaming/gambling activity, impulsivity, emotion dysregulation, and risk of social network and Internet use related to IGD and OGD severity. The severity of OGD and IGD was operationalized as the number of DSM-5 criteria met for each disorder. The maximum likelihood estimator was used, and all parameters were freely estimated, allowing the model to optimally reproduce the observed covariance structure. Given the large number of subscales across the questionnaires employed in the study, total scores were used for each measure, and the Global Severity Index (GSI) of the SCL-90-R was used to represent overall psychopathology. Psychological distress was modeled as an outcome due to its broad, state-like nature and, from a clinical perspective, its conceptual similarity to IGD and OGD as a consequence of behavioral dysregulation. In contrast, mediator constructs such as impulsivity or emotion dysregulation are more stable over time. An initial model was estimated including all plausible direct and indirect effects among the study variables, as well as covariances among exogenous variables, reflecting the exploratory nature of the study. No additional covariates were included. Non-significant parameters were subsequently removed to yield a more parsimonious and interpretable final model. Model fit was evaluated using standard indices (Barret, 2007): the chi-square test (χ^2^, with non-significant values indicating good fit), the Standardized Root Mean Square Residual (SRMR, with values ≤ 0.10 for adequate fitting), the Root Mean Square Error of Approximation (RMSEA, with values ≤ 0.08 interpreted as adequate), the Comparative Fit Index (CFI ≥ 0.90 interpreted as adequate fit) and the Tucker-Lewis Index (TLI, with values ≥ 0. indicating satisfactory model fit). Model fit indices were reported for this final model.

## Results

### Demographic Characteristics of the Sample

The study sample included 273 first-year undergraduate students, of whom 180 were women (65.9%) and 93 were men (34.1%), with a mean age of 19.5 years (SD = 1.9). The majority were born in Spain (n = 256, 93.8%). Regarding socioeconomic background, the distribution of the social position index was as follows: high (n = 42, 15.4%), medium–high (n = 85, 31.1%), medium (n = 46, 16.8%), medium–low (n = 66, 24.2%), and low (n = 34, 12.5%).

### Presence of IGD and OGD Severity Groups

**Fig. 1:**
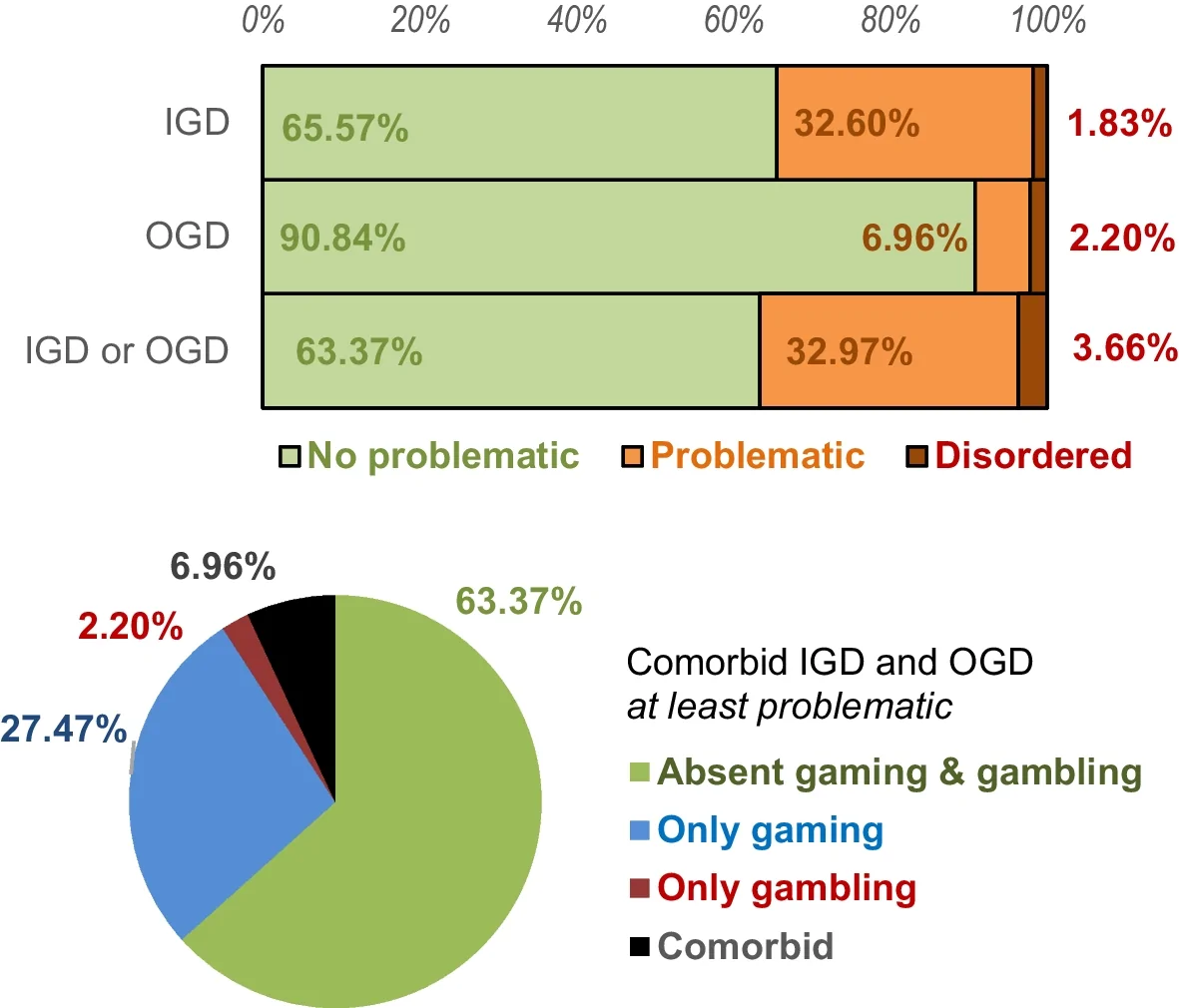
Presence of IGD and OGD severity groups. Note. IGD: Internet gaming disorder. OGD: Online gambling disorder. Problematic IGD: between 1 to 4 DSM-5 criteria. Problematic OGD: between 1 to 3 DSM-5 criteria

Table 1 presents the proportion estimates for IGD and OGD (see Fig. 1). For IGD, 32.6% of participants endorsed one to four DSM-5 criteria, thus meeting the threshold for problematic use, whereas 1.8% met the full diagnostic criteria for disordered IGD. Regarding OGD, 7.0% of participants reported problematic behavior (one to three DSM-5 criteria), and 2.2% satisfied the diagnostic threshold for disordered OGD. When considering the presence of DSM-5 criteria for either IGD or OGD, 33.0% of the sample endorsed problematic behaviors, and 3.7% met the diagnostic criteria for at least one of these conditions. Finally, concerning comorbid profiles (concurrent IGD and OGD), 7.0% of participants reported problematic levels in both addictive conditions, whereas only 0.4% fulfilled the complete DSM-5 diagnostic criteria for both IGD and OGD simultaneously.

**Table 1:** Presence of IGD and OGD severity groups

		n	%	95% CI	
Internet gaming	Absent or no problematic	179	65.57%	59.93%	71.20%
	Problematic	89	32.60%	27.04%	38.16%
	Disordered	5	1.83%	0.24%	3.42%
Online gambling	Absent or no problematic	248	90.84%	87.42%	94.26%
	Problematic	19	6.96%	3.94%	9.98%
	Disordered	6	2.20%	0.46%	3.94%
IGD or OGD	Absent or no problematic	173	63.37%	57.65%	69.09%
	Problematic IGD or OGD	90	32.97%	27.39%	38.54%
	Disordered IGD or OGD	10	3.66%	1.43%	5.89%
Comorbidity: at least problematic	Absent or no problematic	173	63.37%	57.65%	69.09%
	Only IGD	75	27.47%	22.18%	32.77%
	Only OGD	6	2.20%	0.46%	3.94%
	Comorbid IGD and OGD	19	6.96%	3.94%	9.98%
Comorbidity: disordered	No comorbid	272	99.63%	98.92%	100.00%
	Comorbid	1	0.37%	0.00%	1.08%

Note. IGD: Internet gaming disorder. OGD: Online gambling disorder. 95% CI: 95% confidence interval

Problematic IGD: between 1 to 4 DSM-5 criteria. Problematic OGD: between 1 to 3 DSM-5 criteria.

**Fig. 2:**
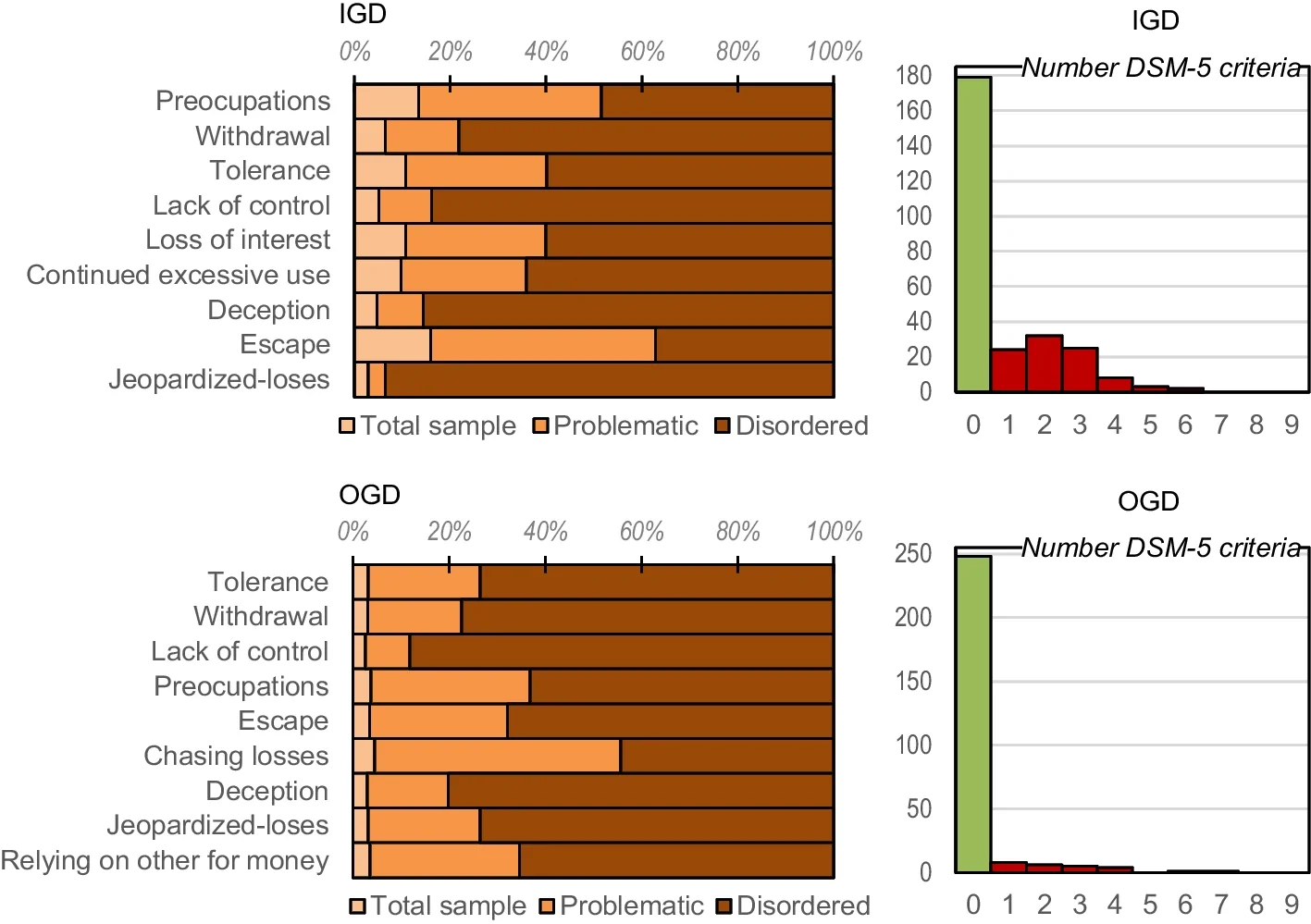
Presence of the DSM-5 criteria for OGD and IGD. Note. IGD: Internet gaming disorder. OGD: Online gambling disorder. Problematic IGD: between 1 to 4 DSM-5 criteria. Problematic OGD: between 1 to 3 DSM-5 criteria

Table 2 displays the proportion of individual DSM-5 criteria for both IGD and OGD across the full sample, as well as among participants classified as problematic or disordered (see Fig. 2). For IGD, the most frequently endorsed criteria in the total sample were escape motivation (use of gaming to relieve negative moods) (17.2%) and preoccupation with Internet gaming (16.5%). These two criteria also showed the highest proportion among participants within the problematic IGD range (endorsed by 50.6% and 47.2%, respectively), followed by continued excessive gaming despite problems (32.6%). Within the disordered IGD group, endorsement rates for all criteria ranged between 40 and 80%, with the most prevalent withdrawal and continued excessive gaming despite negative consequences.

**Table 2:** Presence of the DSM-5 criteria for OGD and IGD

	Total N = 273		Problematic IGD N = 89		Disordered IGD N = 5	
Criteria for IGD	n	%	n	%	n	%
Preoccupation with Internet games	45	16.48%	42	47.19%	3	60.00%
Withdrawal (irritable-anxious when stop gaming)	18	6.59%	14	15.73%	4	80.00%
Tolerance (need of gaming for more time)	29	10.66%	26	29.55%	3	60.00%
Repeated unsuccessful attempts to control gaming	10	3.66%	7	7.87%	3	60.00%
Loss of interest in other activities	29	10.62%	26	29.21%	3	60.00%
Continued excessive gaming despite problems	33	12.09%	29	32.58%	4	80.00%
Deception (lying others about gaming activity)	6	2.20%	4	4.49%	2	40.00%
Escape (use gaming to relieve negative moods)	47	17.22%	45	50.56%	2	40.00%
Jeopardized or losing relationships/opportunities	5	1.84%	2	2.27%	3	60.00%
	Total N = 273		Problematic OGD N = 19		Disordered OGD N = 6	
Criteria for OGD	n	%	n	%	n	%
Tolerance (need of gambling with more money)	6	2.21%	3	15.79%	3	50.00%
Withdrawal (irritable-anxious when stop gambling)	9	3.31%	4	21.05%	5	83.33%
Repeated unsuccessful attempts to control gambling	8	2.94%	2	10.53%	6	100.00%
Preoccupation with online gambling	8	2.95%	5	26.32%	3	50.00%
Escape (gambling to relieve negative moods)	7	2.57%	4	21.05%	3	50.00%
Chasing losses	14	5.17%	11	57.89%	3	50.00%
Deception (lying others about gambling activity)	5	1.85%	2	10.53%	3	50.00%
Jeopardized or losing relationships/opportunities	2	0.74%	1	5.26%	1	16.67%
Relying on others to provide money for gambling	5	1.84%	3	15.79%	2	33.33%

Note. IGD: Internet gaming disorder. OGD: Online gambling disorder

Problematic IGD: between 1 to 4 DSM-5 criteria. Problematic OGD: between 1 to 3 DSM-5 criteria.

For OGD, the most prevalent criterion in the total sample was chasing losses (5.2%), followed by withdrawal (3.3%). Among participants with problematic OGD, chasing losses was again the most frequently endorsed criterion (57.9%). Within the disordered OGD group, repeated unsuccessful attempts to control gambling was universally endorsed (100%), followed by withdrawal (83.3%).

### Factors Related to the Presence of IGD and OGD

Table 3 shows the results of the comparisons across IGD and OGD groups. Significant gender differences emerged in both IGD and OGD, with men overrepresented in the problematic–disordered groups. No differences were observed for age, country of origin, or socioeconomic status.

**Table 3:** Comparison between the groups

	IGD						OGD					
	Non problematic N = 179		Problematic- disordered N = 94				Non problematic N = 248		Problematic- disordered N = 25			
	n	%	n	%	p	C-V	n	%	n	%	p	C-V
GenderWomen	148	82.2%	32	17.8%	<.001*	.488^†^	174	96.7%	6	3.3%	<.001*	.281^†^
Men	31	33.3%	62	66.7%			74	79.6%	19	20.4%		
OriginSpain	169	66.0%	87	34.0%	.546	.037	233	91.0%	23	9.0%	.700	.023
Other	10	58.8%	7	41.2%			15	88.2%	2	11.8%		
Social indexHigh	33	78.6%	9	21.4%	.148	.158	39	92.9%	3	7.1%	.973	.043
Mean-high	58	68.2%	27	31.8%			77	90.6%	8	9.4%		
Mean	27	58.7%	19	41.3%			42	91.3%	4	8.7%		
Mean-low	43	65.2%	23	34.8%			60	90.9%	6	9.1%		
Low	18	52.9%	16	47.1%			30	88.2%	4	11.8%		
	Mean	SD	Mean	SD	p	|d|	Mean	SD	Mean	SD	p	|d|
Age (years-old)	19.50	1.85	19.51	1.89	.974	0.00	19.50	1.89	19.60	1.63	.791	0.06
RASNI Addictive symptoms	21.01	4.06	23.16	4.09	<.001*	0.53^†^	21.59	4.15	23.32	4.34	.049*	0.41
RASNI Social use	17.67	3.83	17.38	3.48	.544	0.08	17.63	3.68	17.04	3.99	.453	0.15
RASNI Freaky	8.21	1.97	11.12	2.82	<.001*	1.20^†^	8.93	2.48	11.96	3.05	<.001*	1.09^†^
RASNI Nomophobia	13.71	2.74	15.95	3.45	<.001*	0.72^†^	14.38	3.15	15.48	3.39	.099	0.34
RASNI Total	60.59	8.50	67.61	10.01	<.001*	0.76^†^	62.52	9.31	67.80	11.51	.009*	0.50^†^
GRCS Expectancies	6.16	3.91	10.54	6.03	<.001*	0.86^†^	6.95	4.47	14.80	6.28	<.001*	1.44^†^
GRCS Illusion of control	5.32	3.05	6.36	3.96	.016*	0.30	5.46	3.19	7.88	4.73	.001*	0.60^†^
GRCS Predictive control	9.21	5.33	13.29	7.62	<.001*	0.62^†^	9.71	5.60	19.60	7.92	<.001*	1.44^†^
GRCS Inability to stop	6.08	3.15	7.48	4.05	.002*	0.38	6.15	2.94	10.64	5.82	<.001*	0.97^†^
GRCS Interpretative bias	6.31	4.23	10.35	7.01	<.001*	0.70^†^	6.97	5.01	14.92	6.85	<.001*	1.32^†^
GRCS Total	33.08	17.51	48.02	23.96	<.001*	0.71^†^	35.24	18.23	67.84	25.44	<.001*	1.47^†^
UPPS-P Lack premeditation	21.25	5.31	25.67	8.08	<.001*	0.65^†^	22.53	6.67	25.20	6.90	.058	0.39
UPPS-P Lack perseveration	20.36	5.10	24.67	7.51	<.001*	0.67^†^	21.66	6.36	23.68	6.24	.130	0.32
UPPS-P Sensation seeking	29.12	8.54	29.11	6.52	.991	0.00	28.68	7.82	33.44	7.41	.004*	0.63^†^
UPPS-P Positive urgency	22.68	7.40	28.21	8.30	<.001*	0.70^†^	23.92	7.88	31.12	8.00	<.001*	0.91^†^
UPPS-P Negative urgency	26.44	6.92	30.64	7.59	<.001*	0.58^†^	27.71	7.53	29.64	6.05	.216	0.28
UPPS-P Total	119.84	22.03	138.30	30.59	<.001*	0.69^†^	124.50	26.40	143.08	24.54	.001*	0.73^†^
DERS Non acceptance emotions	14.20	5.50	15.09	5.88	.216	0.16	14.62	5.67	13.36	5.26	.289	0.23
DERS Diff. directed behaviours	15.56	4.22	17.50	4.43	<.001*	0.45	16.31	4.37	15.48	4.52	.370	0.19
DERS Impulse control difficulties	13.46	5.10	15.74	5.98	.001*	0.41	14.19	5.60	14.84	4.78	.573	0.13
DERS Lack emotional awareness	14.31	4.86	15.73	5.13	.025*	0.29	14.73	5.02	15.52	4.73	.449	0.16
DERS Limited access emotions	19.26	6.53	22.35	7.34	<.001*	0.45	20.30	7.01	20.56	6.61	.858	0.04
DERS Lack emotional clarity	12.12	4.12	12.22	4.83	.849	0.02	12.15	4.40	12.24	4.17	.918	0.02
DERS Total	88.90	21.33	98.64	24.14	.001*	0.43	92.28	22.86	92.00	22.30	.954	0.01
SCL-90R Somatization	1.12	0.71	1.20	0.69	.357	0.12	1.14	0.71	1.27	0.72	.352	0.19
SCL-90R Obsessive/compulsive	1.45	0.84	1.27	0.93	.124	0.19	1.38	0.88	1.42	0.86	.858	0.04
SCL-90R Interpersonal sensitivity	1.49	0.91	1.42	0.81	.513	0.09	1.46	0.89	1.52	0.76	.755	0.07
SCL-90R Depressive	1.31	0.82	1.22	0.83	.414	0.10	1.27	0.83	1.35	0.79	.668	0.09
SCL-90R Anxiety	1.09	0.74	1.08	0.59	.887	0.02	1.09	0.70	1.12	0.56	.821	0.05
SCL-90R Hostility	0.86	0.75	1.20	0.78	.001*	0.44	0.93	0.76	1.46	0.79	.001*	0.69^†^
SCL-90R Phobic anxiety	0.74	0.70	0.76	0.61	.766	0.04	0.74	0.68	0.78	0.59	.766	0.07
SCL-90R Paranoid Ideation	1.19	0.81	1.33	0.72	.165	0.18	1.20	0.79	1.59	0.66	.019*	0.53^†^
SCL-90R Psychotic	0.78	0.72	0.74	0.73	.691	0.05	0.75	0.71	0.86	0.83	.471	0.14
SCL-90R GSI	1.14	0.66	1.14	0.60	.982	0.00	1.13	0.65	1.26	0.58	.329	0.21
SCL-90R PST	52.69	21.47	50.74	21.07	.474	0.09	51.55	21.51	56.72	19.02	.248	0.25
SCL-90R PSDI	1.83	0.49	1.94	0.42	.069	0.24	1.86	0.47	1.99	0.48	.195	0.27

Note. SD: standard deviation. C-V: Cramer’s-V. |d|: Cohen’s-d coefficient. *Bold: significant comparison. ^†^Bold: effect size into the mild/moderate to high/large. IGD: Internet gaming disorder. OGD: Online gambling disorder. RASNI: Scale of Risk of Addiction to Social Networks and Internet (ERA-RSI in Spanish version). GRCS: Gambling Related Cognitions Scale. UPPS-P: Impulsive Behavior Scale. DERS: Difficulties in Emotion Regulation Scale. SCL-90R: Symptom Checklist-90-Revised

**Fig. 3:**
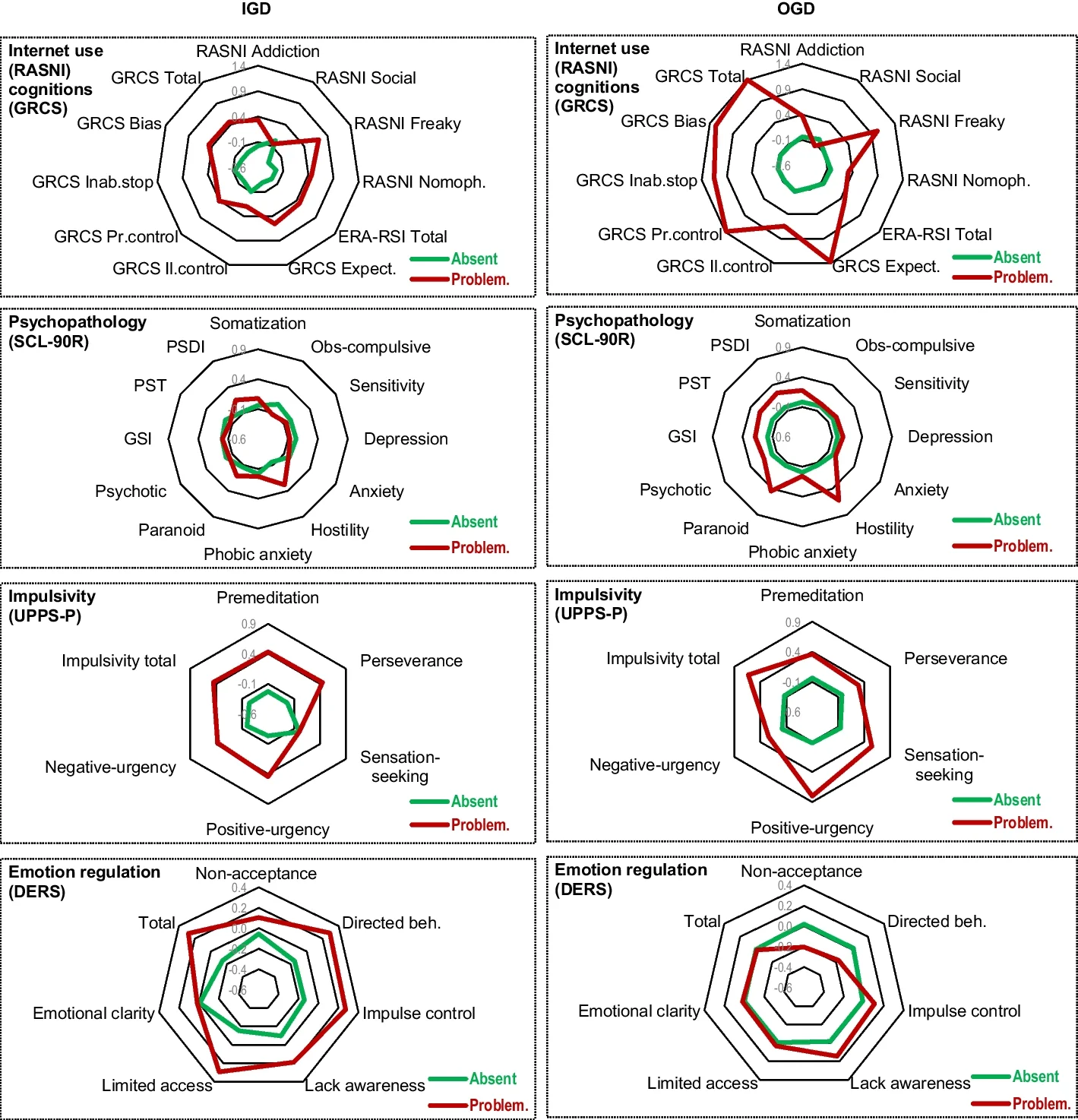
Radar-chart with the clinical composition of the groups. Note. IGD: Internet gaming disorder. OGD: Online gambling disorder. RASNI: Scale of Risk of Addiction to Social Networks and Internet (ERA-RSI in Spanish version). GRCS: Gambling Related Cognitions Scale. UPPS-P: Impulsive Behavior Scale. DERS: Difficulties in Emotion Regulation Scale. SCL-90R: Symptom Checklist-90-Revised

In relation to social networks and Internet use, participants with problematic–disordered IGD scored higher on all RASNI scales except social use, whereas those with problematic–disordered OGD obtained higher scores in addictive symptoms, freaky use, and the total scale. Gambling/gaming-related cognitions (GRCS) were consistently elevated across all subscales and total scores in both IGD and OGD. Regarding impulsivity, problematic–disordered IGD participants scored higher on all UPPS-P dimensions except sensation seeking, while problematic–disordered OGD participants showed higher scores in sensation seeking, positive urgency, and the total scale. Emotion regulation deficits (DERS) were greater among problematic–disordered IGD participants—except for non-acceptance of emotions and lack of emotional clarity—whereas no significant group differences emerged for OGD. Finally, psychopathological symptoms (SCL-90-R) revealed limited differences: higher hostility in problematic–disordered IGD, and higher hostility and paranoid ideation in problematic–disordered OGD. Figure 3 presents radar charts summarizing the differences between groups across all the scales analyzed in the study. For ease of interpretation, Z-standardized means are displayed, providing a concise visual summary of the relative profiles of problematic–disordered versus non-problematic participants.

### Path analysis

**Fig. 4:**
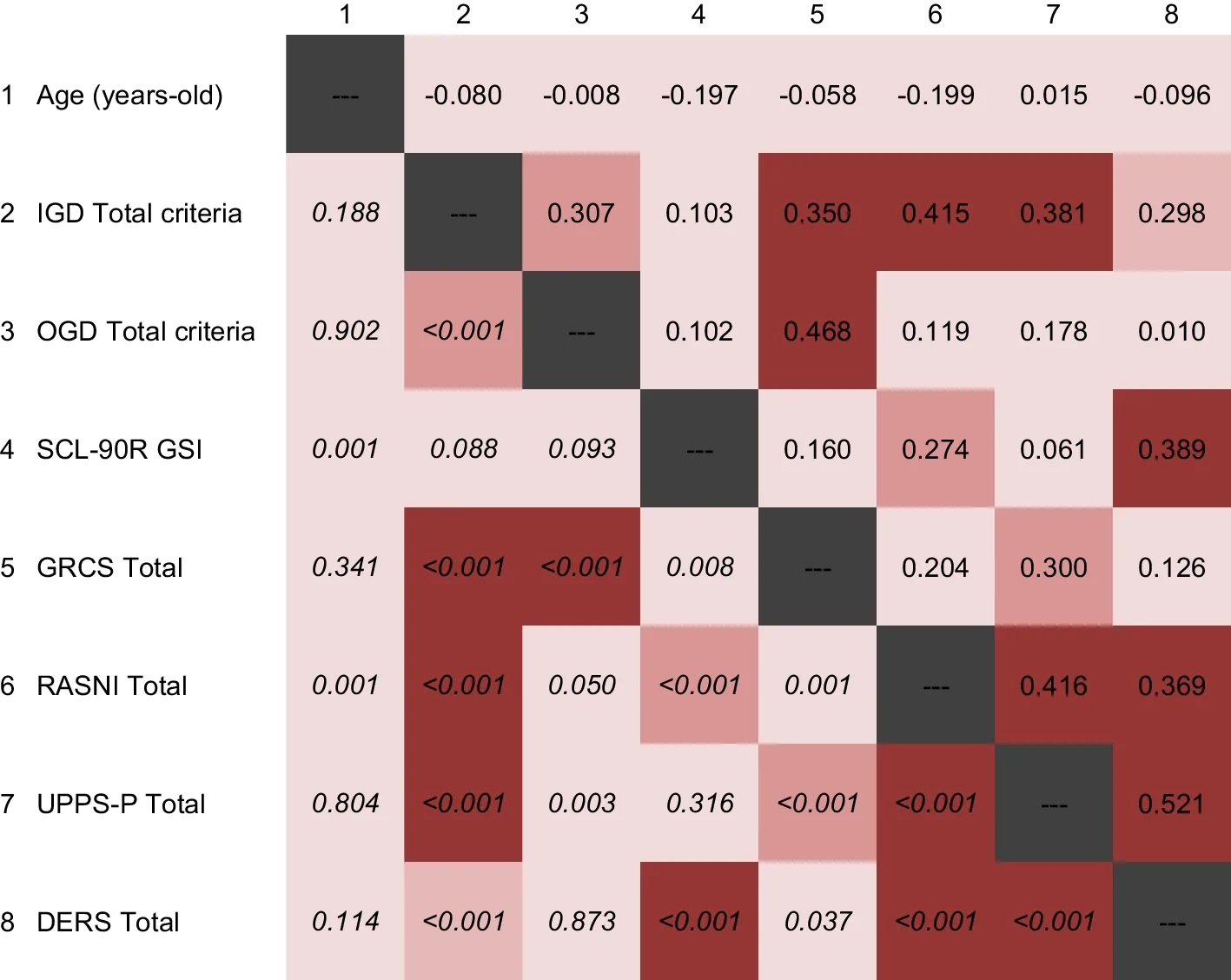
Heatmap of the correlation matrix for the variables analyzed in the SEM. Note. Upper diagonal part (normal font): correlation coefficients. Lower diagonal part (italics font): p-vlaues. IGD: Internet gaming disorder. OGD: Online gambling disorder. SCL-90R: Symptom Checklist-90-Revised. GRCS: Gambling Related Cognitions Scale. RASNI: Scale of Risk of Addiction to Social Networks and Internet (ERA-RSI in Spanish version). UPPS-P: Impulsive Behavior Scale. DERS: Difficulties in Emotion Regulation Scale

Figure 4 displays the heatmap for the correlation matrix with the bivariate associations for the variables included in the SEM (the upper diagonal part of the plot contains the correlation coefficients, and the lower diagonal part shows the level of statistical significance).

**Fig. 5:**
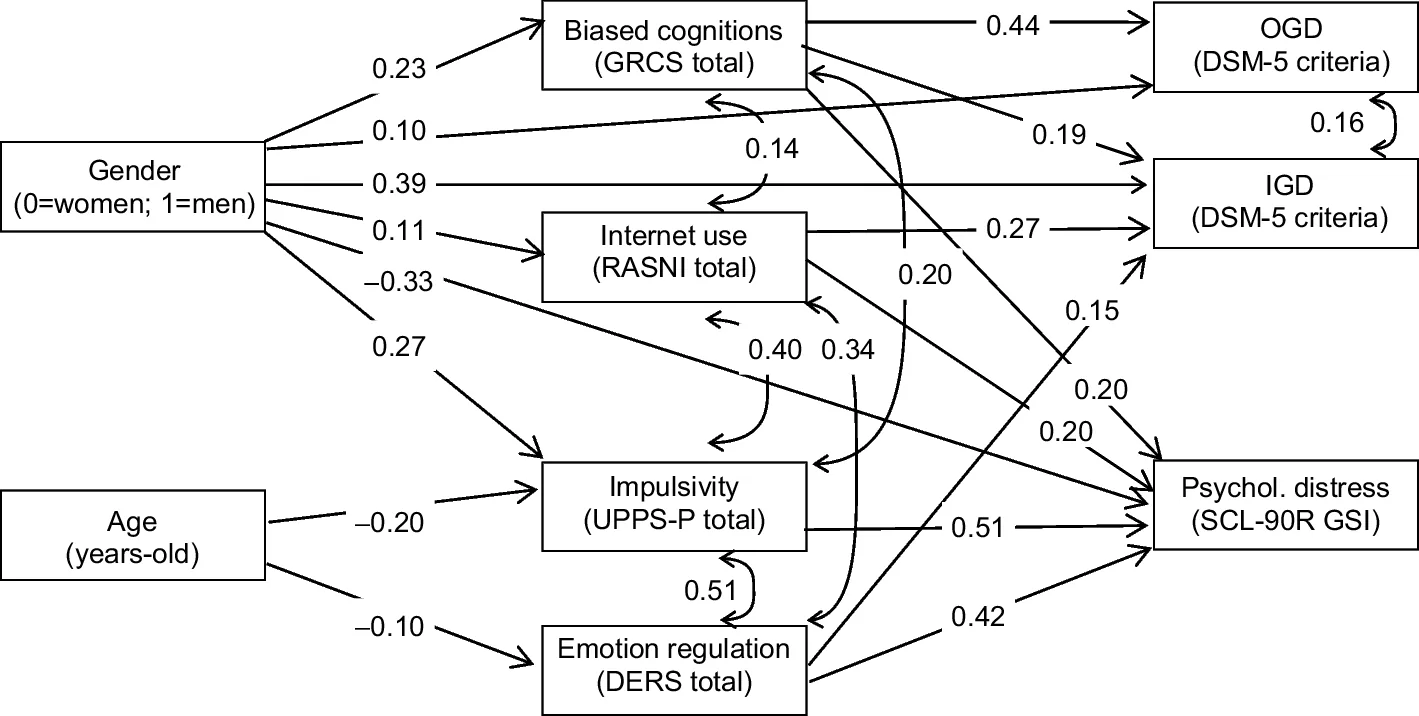
Path-diagram with the standardized coefficients obtained in the SEM. Note. OGD: online gambling disorder. IGD: Internet gaming disorder. RASNI: Scale of Risk of Addiction to Social Networks and Internet (ERA-RSI in Spanish version). GRCS: Gambling Related Cognitions Scale. UPPS-P: Impulsive Behavior Scale. DERS: Difficulties in Emotion Regulation Scale. SCL-90R: Symptom Checklist-90-Revised

Figure 5 displays the path-diagram with the standardized coefficients obtained in the SEM (complete results are available in Table 4). Adequate goodness-of-fit was obtained: χ^2^ = 17.87 (p = 0.163); RMSEA = 0.037 (95% confidence interval: 0.001 to 0.075), CFI = 0.991, TLI = 0.976 and SRMR = 0.044. The global predictive capacity of the model (as measured with the coefficient of determination) was CD = 0.389.

**Table 4:** Complete results obtained in the SEM

Direct effects		Coeff	SE	z-stat	p	95%CI coefficient	
RASNI total	Gender	2.3079	1.1216	2.06	.040	0.1096	4.5063
	Age	−1.0413	0.2779	−3.75	<.001	−1.5859	−0.4967
GRCS total	Gender	10.2396	2.6244	3.9	<.001	5.0958	15.3834
UPPS-P total	Age	14.6393	2.7796	5.27	<.001	9.1914	20.0873
Gaming	RASNI total	0.0369	0.0069	5.33	<.001	0.0233	0.0505
	GRCS total	0.0118	0.0030	3.86	<.001	0.0058	0.0177
	DERS total	0.0086	0.0029	2.97	.003	0.0029	0.0142
	Gender	1.0545	0.1340	7.87	<.001	0.7919	1.3171
Gambling	GRCS total	0.0185	0.0023	8.15	<.001	0.0141	0.0230
	Gender	0.1886	0.1013	1.86	.063	−0.0099	0.3871
SCL-90R GSI	RASNI total	0.0137	0.0037	3.7	<.001	0.0064	0.0209
	GRCS total	0.0061	0.0016	3.85	<.001	0.0030	0.0093
	UPPS-P total	−0.0049	0.0015	−3.17	.002	−0.0079	−0.0019
	DERS total	0.0119	0.0017	7.16	<.001	0.0087	0.0152
	Gender	−0.4531	0.0709	−6.39	<.001	−0.5922	−0.3141
DERS total	Age	−1.2552	0.6303	−1.99	.046	−2.4905	−0.0198
Indirect effects		Coeff	SE	z-stat	p	95%CI coefficient	
Gaming	Gender	0.2056	0.0641	3.21	.001	0.0800	0.3313
	Age	−0.0492	0.0142	−3.47	.001	−0.0770	−0.0214
Gambling	Gender	0.1897	0.0539	3.52	<.001	0.0840	0.2953
SCL-90R GSI	Gender	0.0230	0.0334	0.69	.491	−0.0425	0.0884
	Age	−0.0292	0.0099	−2.96	.003	−0.0485	−0.0099
Total effects		Coeff	SE	z-stat	p	95%CI coefficient	
RASNI total	Gender	2.3079	1.1216	2.06	.040	0.1096	4.5063
	Age	−1.0413	0.2779	−3.75	<.001	−1.5859	−0.4967
GRCS total	Gender	10.2396	2.6244	3.9	<.001	5.0958	15.3834
UPPS-P total	Age	14.6393	2.7796	5.27	<.001	9.1914	20.0873
Gaming	RASNI total	0.0369	0.0069	5.33	<.001	0.0233	0.0505
	GRCS total	0.0118	0.0030	3.86	<.001	0.0058	0.0177
	DERS total	0.0086	0.0029	2.97	.003	0.0029	0.0142
	Gender	1.2601	0.1415	8.91	<.001	0.9828	1.5374
	Age	−0.0492	0.0142	−3.47	.001	−0.0770	−0.0214
Gambling	GRCS total	0.0185	0.0023	8.15	<.001	0.0141	0.0230
	Gender	0.3783	0.1099	3.44	.001	0.1629	0.5937
SCL-90R GSI	RASNI total	0.0137	0.0037	3.7	<.001	0.0064	0.0209
	GRCS total	0.0061	0.0016	3.85	<.001	0.0030	0.0093
	UPPS-P total	−0.0049	0.0015	−3.17	.002	−0.0079	−0.0019
	DERS total	0.0119	0.0017	7.16	<.001	0.0087	0.0152
	Gender	−0.4301	0.0707	−6.09	<.001	−0.5687	−0.2916
	Age	−0.0292	0.0099	−2.96	.003	−0.0485	−0.0099
DERS total	Age	−1.2552	0.6303	−1.99	.046	−2.4905	−0.0198

Note. SD: standard deviation. C-V: Cramer’s-V. |d|: Cohen’s-d coefficient. *Bold: significant comparison. ^†^Bold: effect size into the mild/moderate to high/large. RASNI: Scale of Risk of Addiction to Social Networks and Internet (ERA-RSI in Spanish version). GRCS: Gambling Related Cognitions Scale. UPPS-P: Impulsive Behavior Scale. DERS: Difficulties in Emotion Regulation Scale. SCL-90R: Symptom Checklist-90-Revised

The results of the SEM indicated that the higher IGD severity was positively predicted by the elevated risk of social networks and Internet use, higher levels of gambling/gaming-related biased cognitions, emotion regulation deficit, and male gender. In contrast, OGD severity was predicted by higher levels of gambling/gambling-related biased cognitions and male gender.

Indirect effects also showed that gender influenced both IGD and OGD severity via the mediating role of biased cognitions and the engagement in social network and Internet: being men increased the likelihood of high GRCS and RASNI scores, which next contributed to increased addiction levels. Age had a negative indirect effect on IGD severity via the mediating role of emotion regulation: younger individuals were more prone to emotion regulation deficit, and this path contributed to increased IGD.

## Discussion

The present study estimated the propontion of IGD and OGD among first-year university students, together with their associated correlates, and examined both the direct and indirect effects of individual psychological vulnerabilities. Problematic IGD affected 34.4% of students and OGD 9.2%, with 7.0% showing comorbid involvement. IGD severity was linked to male gender, social network/Internet use, cognitive biases, and emotion regulation difficulties. OGD severity was primarily associated with male gender and gambling-related cognitive biases, with age and gender effects mediated by psychological vulnerabilities.

Several noteworthy findings emerged from this study. Regarding proportion estimates, approximately one-third of the participants endorsed at least one DSM-5 criterion for IGD or OGD, and 3.7% met the full diagnostic threshold for at least one of these conditions. These rates fall within the range published in current systematic reviews (Männikkö et al., 2020; Mihara & Higuchi, 2017; Montag et al., 2024; Nowak, 2018), suggesting that problematic gaming and gambling behaviors are a significant concern among young adults in academic contexts. Importantly, while comorbid endorsement of at least problematic symptoms for IGD plus OGD was relatively frequent (7%), the proportion of students meeting full diagnostic criteria for both conditions was negligible (0.4%). This finding underscores that, although symptom overlap is not uncommon (Hofstedt & Söderpalm Gordh, 2024), the progression to severe comorbid behavioral addiction remains rare at this developmental stage.

Differences emerged in the endorsement of specific DSM-5 criteria depending on whether participants exhibited IGD- or OGD-related symptoms. Previous studies observed that both clinical conditions share features of behavioral addiction (Karlsson et al., 2019; Ko et al., 2020; Mallorquí-Bagué et al., 2017), including overlaps in neural activity, cognitive functioning, and other socio-psychological features (Vaccaro & Potenza, 2019). The relative prominence of precise criteria depending on the specific online addiction identified in this work reflects differences in triggering motivations and exacerbation mechanisms.

For IGD, the prominence of escape motivation and preoccupation observed in both the total sample and among problematic users indicates that gaming is frequently endorsed as a strategy for mood regulation or stress relief, consistent with prior research highlighting the role of mood modification in the onset and primary stages of this behavioral addiction among young age individuals (Bäcklund et al., 2022; Brand et al., 2020; Király et al., 2015; Marques et al., 2023; Paulus et al., 2018; Sinha et al., 2024). But given the limited specificity of escape-related criteria, particularly in young populations, these findings likely reflect a common feature of gaming engagement rather than a core mechanism of disordered gaming. Accordingly, mood modification may be more relevant to early or subthreshold manifestations of gaming-related difficulties than to the differentiation of IGD as a distinct clinical condition. The high endorsement of withdrawal and continued excessive gaming among individuals who met full criteria for IGD indicates that, as severity increases, users may experience more pronounced functional impairments and psychological withdrawal symptoms, reflecting a shift from coping-driven to compulsive behavior (Fernandez et al., 2020; Kaptsis et al., 2016; King et al., 2017).

In contrast, OGD appears to be characterized by patterns more closely linked to the pressure and financial contingencies of gambling. Chasing losses emerged as the most frequently endorsed criterion across the total sample and among problematic gamblers, reflecting the reinforcing cycle of risk and reward inherent in gambling behavior, particularly during its early stages (Banerjee et al., 2023; Zhang & Clark, 2020). Withdrawal symptoms were also highly prevalent, consistent with prior studies emphasizing the strong discriminatory power of this criterion in identifying gambling severity (Lucas et al., 2024; Sleczka et al., 2015). But the secondary prominence of withdrawal relative to chasing losses observed in the present study suggests that, particularly in the early stages of the disorder, OGD may be driven more by reinforcement-based mechanisms than by emotion-regulation motives. Moreover, the near-universal endorsement of unsuccessful attempts to control gambling among disordered OGD participants provides strong evidence of the compulsive dimension of the disorder. This criterion captures the persistence of gambling behavior despite the individual’s explicit intention and repeated efforts to cease. At advanced stages of gambling disorder, clinical models have proposed that gambling behavior may become increasingly resistant to executive regulatory mechanisms, potentially resulting in continued engagement despite acknowledged adverse consequences and sustained motivation to quit. Prior clinical and neurobiological research has described a shift from goal-directed or recreational gambling toward behavioral patterns characterized by strong urges, habitual repetition, and diminished regulatory control, which are associated with greater functional impairments (e.g., financial losses, relationship conflict, occupational or academic decline, and emotional distress) (Solly et al., 2025). Consistent with these conceptual models, findings from independent neurobiological studies have reported frontostriatal alterations (such as changes in dopaminergic signaling in the ventral striatum, altered limbic reinforcement processing, and reduced prefrontal serotonergic modulation) that have been associated with impairments in reward processing, decision-making, and cognitive flexibility in gambling disorder (Figee et al., 2016; van Timmeren et al., 2018). Importantly, these neurobiological mechanisms were not assessed in the present study and are discussed here solely to provide theoretical context for the observed behavioral patterns.

The results obtained in the bivariate group comparisons provided valuable insights into underlying individual vulnerabilities and suggested potential mechanisms that may increase susceptibility to IGD and OGD. Gender differences emerged for both addictive behaviors: men were overrepresented in the problematic–disordered groups. Notably, no significant differences were observed between groups in terms of socioeconomic background or country of origin, suggesting that these demographic factors may have limited influence within university populations, while psychological and cognitive mechanisms may play a more decisive role in distinguishing students at risk for behavioral addictions. Regarding the other variables, as expected, participants in the problematic gaming–gambling group exhibited greater dysfunction, indicating more pronounced impairments across psychological, behavioral, and functional domains.

SEM analysis examined the explanatory mechanisms contributing to the severity of IGD and OGD among first-year university students. IGD severity was directly associated to male gender, biased cognitions (as measured with the GRCS), higher engagement in Internet and social network use, and emotion regulation deficits; OGD severity, in contrast, was directly related to male gender and biased cognitions. These results underscore the presence of both common and disorder-specific etiological direct pathways among these addictions. Specifically, male gender and cognitive distortions were identified as shared vulnerabilities, highlighting their potential role as transdiagnostic risk factors across these behavioral addictions. These results are consistent with previous studies that emphasized the role of gender (Dellosa & Browne, 2024; Dong & Potenza, 2022; Karlsson et al., 2019) and irrational beliefs (Armstrong et al., 2020; Wu et al., 2018) not only to the initiation but also to the escalation and persistence of the disorders. Men may be more prone to these addictive behaviors due to a combination of psycho-sociocultural influences that increase engagement in online gaming or gambling: behavioral preferences for competitive and reward-driven online activities, cognitive tendencies toward risk-taking, higher risk tolerance and lower perception of negative consequences of excessive online activity, sociocultural reinforcement of mastery and status (e.g. peer dynamics and cultural norms normalize gaming and gambling as masculine activities), and targeted marketing and reward-driven game designs. Evidence from neurobiological research has suggested that sex differences may play a role in vulnerability to Internet-related addictive behaviors, with some studies reporting sex-specific patterns of activation in brain regions involved in reward processing and decision-making. In particular, neural circuits related to attention, executive functioning, and sensorimotor integration have been shown to exhibit differential engagement across sexes (Chen et al., 2018). These findings point to potential functional differences in neural substrates that have been implicated in both IGD and OGD in independent neurobiological research (Marraudino et al., 2022). However, such mechanisms were not directly examined in the present study and are discussed here as a broader theoretical framework rather than as evidence derived from the current data. Regarding irrational beliefs (such as illusions of control, overestimation of winning probabilities, or misattribution of outcomes) their role in exacerbating risk-taking and compulsive behaviors has been well established in gambling disorder (Armstrong et al., 2020). By contrast, the nature and specificity of comparable cognitive distortions in gaming remain less clearly defined. Consequently, when gambling-related measures are applied to gaming populations, the resulting findings are better understood as reflecting partially overlapping or adjacent cognitive processes (e.g., performance- or skill-related beliefs), rather than direct analogues of gambling-specific distortions.

The convergences observed between IGD and OGD are also consistent with current frameworks of Internet-use disorders, such as the “Interaction of Person-Affect-Cognition-Execution” (I-PACE) model (Brand et al., 2016). This approach conceptualizes the onset and progression of Internet addictive behaviors as resulting from dynamic interactions among predisposing factors, moderators, and mediators, along with impaired executive functioning and compromised decision-making, which together contribute to the emergence and maintenance of these behaviors. Among young university students, the I-PACE model suggests that affective processes (such as stress, anxiety, or depressed mood) can drive Internet use as a maladaptive coping strategy, offering temporary relief but reinforcing reliance on online activities. Cognitive distortions, including overestimating social or academic benefits and perceived control over outcomes, further entrench engagement and impair self-regulation. Coupled with still-maturing executive functions, particularly inhibitory control and decision-making, these interacting factors increase vulnerability to compulsive Internet behaviors and contribute to the heterogeneity observed in Internet addiction severity within this population (Brand et al., 2019; Wegmann & Brand, 2021).

The distinct profiles observed in IGD and OGD can be examined from multiple perspectives. From a neuroscientific standpoint, prior research has suggested that these conditions may involve partially divergent neurocognitive systems. In IGD, elevated impulsivity (excluding sensation seeking) and broad emotion regulation difficulties suggest dysregulation of prefrontal–limbic circuits (Khor et al., 2024). Studies have observed that patients with IGD show dysfunctional dorsomedial prefrontal cortex mediating abnormal emotional influences on response inhibition, expressed by increased activation of widespread brain regions (including prefrontal, motor-sensory, parietal, occipital, insula, right ventral striatum, and striatal regions) (Shin et al., 2021; Turel et al., 2021; Wu et al., 2020). Excessive use of screens among IGD patients has also been related to the activation of many brain regions associated with cognitive, motor, and sensory function, and these processes have not been directly involved in other forms of addiction (Weinstein & Lejoyeux, 2020). In short, deficits in dorsolateral and ventromedial prefrontal cortex functioning impair top-down inhibitory control and cognitive reappraisal, while hyperactivity in the amygdala and insula amplifies negative affect, facilitating the use of gaming as an avoidance-based coping strategy that may escalate into compulsive engagement. In OGD, the prominence of sensation seeking and positive urgency reflects hypersensitivity of mesocorticolimbic reward systems alongside reduced prefrontal modulation (orbitofrontal cortex, anterior cingulate) (Balodis & Potenza, 2016; Fauth-Bühler et al., 2017), heightened dopaminergic reactivity in the ventral striatum and nucleus accumbens (Kayser, 2019; Zack et al., 2020), which reinforces reward-related learning and promotes goal-directed behavior (Potenza, 2014; Potenza et al., 2019). Furthermore, evidence suggests that distorted gambling-related cognitions are underpinned by aberrant activity within fronto-striatal circuits, fostering expectancy biases and impairments in decision-making processes, thereby perpetuating persistent gambling behavior despite adverse consequences (Armstrong et al., 2020).

The observed divergences between IGD and OGD identified in the bivariate comparisons and the SEM can also be understood considering distinct psychological mechanisms underpinning each condition. In IGD, individuals with poor self-control and limited emotional regulation skills may resort to gaming as an accessible and effective means of immersion and social motivation (Estupiñá et al., 2024; Wang & Cheng, 2022), but also for escaping stress, negative mood, or interpersonal difficulties (Sinha et al., 2024). Over time, this avoidance-based coping fosters increasing preoccupation, functional impairment, and ultimately compulsive use (Demetrovics et al., 2022). In contrast, OGD appears to be more strongly shaped by motivational and cognitive factors associated with reward-seeking (Wegmann et al., 2025), particularly at the first stages of the disorder. High levels of sensation seeking and positive urgency indicate a propensity to pursue stimulation and take risks when experiencing heightened positive affect, and this creates vulnerability to the reinforcing contingencies of gambling (wins, near-misses, and variable reward schedules). This evidence is also consistent with the recent viewpoint about the I-PACE model, that postulates that although both processes are relevant during the entire course of the disorder and a complete shift from gratification to compensation is observed (Brand et al., 2025a, 2025b), the gratifications experienced during gambling could lead to positive reinforcement and decreased control over the activity at the early stages of the disease, and compensation of negative emotions experienced as negative reinforcements should be more intense at later stages (Brand et al., 2019). Moreover, gambling-related cognitive distortions (such as illusions of control or overestimation of winning probabilities) further strengthen engagement and sustain behavior despite accumulating losses. A current study carried out with a sample of young university students (between ages of 18 and 26) observed the predictive capacity of cognitive distortions for the gambling addiction, with this relationship mediated by the maximum amount of money wagered (Esparza-Reig et al., 2023). Studies have also related gambling motives with the development of erroneous beliefs (Mathieu et al., 2020), and these combined factors have been observed as direct and indirect predictors in the development of gambling severity (Ciccarelli et al., 2017).

Finally, the indirect effects observed in the path analysis further suggest age (developmental) specific mechanisms: younger age predicted IGD severity through greater emotion regulation difficulties. The results of this work for age are consistent with prior empirical studies that related young age with higher attentional impulsivity and depressive problems, which acted as mediators of the increased risk of Internet use (Marzilli et al., 2020). In early adulthood, the ongoing maturation of executive functions and emotion regulation may leave younger individuals particularly vulnerable to impulsivity and difficulties managing negative affect, increasing their susceptibility to Internet- and gaming-related addictive behaviors. It has been observed that difficulties in response inhibition and dysfunction within the inhibitory control network (that may be observed in younger adult populations and manifested as altered dorsomedial prefrontal cortex activity) may mediate atypical emotional influences on inhibitory control and engagement in Internet gaming (Shin et al., 2021).

### Limitations and Strengths

Several limitations should be acknowledged. The cross-sectional design prevents causal inferences regarding the observed associations. Secondly, the sample was drawn from a single university, which may limit the generalizability of the findings to broader student populations or other cultural contexts. Thirdly, although the study included a comprehensive assessment of cognitive, emotional, and behavioral variables, other relevant factors such as peer influences, family environment, or genetic predispositions were not examined. Finally, the use of the GRCS represents a limitation of the present study for the measure of the cognitive biases among IGD, as this instrument was not specifically designed to assess gaming-related cognitive distortions and the interpretability of the questionnaire scores is necessarily constrained. Nevertheless, given the increasing convergence between gambling and gaming environments, particularly through gamblified mechanics, the inclusion of the GRCS offers exploratory value by allowing the examination of gambling-related cognitive distortions in young individuals.

Future research should address these limitations by adopting longitudinal designs that can clarify the temporal and causal relationships among cognitive biases, emotion regulation deficits, impulsivity, and the development of IGD and OGD. Empirical studies could further test the causal impact of cognitive distortions and emotion regulation on these addictive behaviors. Moreover, it would be valuable to explore protective factors (such as resilience, social support, and adaptive coping strategies) that may mitigate risk among university students. Expanding research into diverse cultural and educational contexts will also be essential to determine the generalizability of these findings and to inform the development of culturally sensitive interventions, in alignment with research priorities identified by the research community (Czakó et al., 2025).

Beyond its limitations, the study also presents several strengths. It relied on a relatively large and homogeneous sample of first-year university students, a group particularly vulnerable to behavioral addictions, for whom the prevalence and patterns of behavioral addictions remain comparatively less explored. The simultaneous examination of IGD and OGD, along with their comorbid presentations, provides a comprehensive view of behavioral addictions in this target population. Furthermore, the use of validated/standardized instruments spanning multiple psychological domains (cognitive biases, impulsivity, emotion regulation, internet and social media use, and psychopathological symptoms) enabled a multidimensional assessment of IGD and OGD correlates.

### Implications

The clinical and preventive implications of these findings are noteworthy. Cognitive biases and emotion regulation difficulties represent promising targets for intervention among young university students. For IGD, strategies aimed at improving emotional awareness and adaptive regulation may help reduce reliance on gaming as a maladaptive coping mechanism. For OGD, cognitive restructuring techniques that challenge beliefs about chance, control, and expected outcomes may be especially beneficial. Given the overrepresentation of men in the problematic–disordered groups, preventive programs in university settings may need to adopt a gender-sensitive approach, with screening and tailored interventions addressing the specific psychological vulnerabilities associated with each condition.

Recent reviews show that mindfulness and cognitive-behavioral interventions can reduce common mental health problems in higher education students, while evidence on setting-based approaches remains limited (Worsley et al., 2022). Studies have also observed long-term effects for preventive interventions addressed to mental unhealth among students in higher education (especially to reduce depression and anxiety symptoms), but shorter-lasting effects for interventions to promote positive mental health (Winzer et al., 2018). The findings of this study highlight the potential value of incorporating brief interventions and psychoeducational programs into university counseling services to support at-risk students early in their academic trajectory. Such approaches may not only provide timely assistance in managing emerging mental health difficulties but also contribute to the prevention of more severe psychological problems over time. In addition, preventive resilience programs (including cognitive approaches, mindfulness-based strategies, skills training, psychoeducation, and coaching) represent a promising avenue to strengthen students’ coping resources and enhance their capacity to adapt to the unique psychosocial and academic challenges of university life (Abulfaraj et al., 2024).

## Conclusion

Taken together, the findings of this work showed that both IGD and OGD are present among university students, with problematic involvement being relatively common and disordered forms less frequent but clinically relevant. While both conditions share core features of behavioral addiction, their clinical patterns and underlying mechanisms diverge. IGD appears to be primarily driven by escape motivation and maladaptive cognitive-emotional processes that can escalate into compulsive use, whereas OGD is more strongly characterized by contingency patterns (such as loss-chasing) and deficits in inhibitory control mechanisms.

These findings underscore the need for early detection and preventive strategies in university settings. Interventions should focus on correcting maladaptive cognitions, enhancing emotion regulation, and tailoring approaches to the specific profiles of IGD and OGD. Addressing these vulnerabilities early in academic life may reduce the risk of progression to more severe behavioral addictions and mitigate their impact on psychological health and academic functioning.

## Data Availability

The datasets analyzed during the study are not publicly available due to patient confidentiality and other ethical reasons, but are available from the corresponding author on reasonable request.
